# Supplementary material for: Are carbon emissions trading and green financial instruments synergistic? -Comprehensive quantitative research based on content analysis
Source: PLoS One. 2024 Mar 7;19(3):e0298601. doi: 10.1371/journal.pone.0298601 (PMC10919601; doi:10.1371/journal.pone.0298601)
Supplement: S1 Data — (ZIP) [file pone.0298601.s001.zip › Data/地级市Z_F工作报告.zip!/░▓╗╒/│n╓▌╩╨/└┤╫╘╣1⁄2╓┌║┼í╛┬φ┐╦╩2╛▌═°í┐_2016.docx]

**一、破难奋进，过去一年取得显著成绩**

  （一）抓重点求突破，发展势头进一步向好。

  预计全年完成地区生产总值1313亿元，增长10%；规上工业增加值610亿元，增长11%；固定资产投资1450亿元，增长16%；财政收入230.3亿元，增长13.1%，其中地方收入完成143.7亿元，增长16.3%；社会消费品零售总额457亿元，增长12.5%；外贸进出口20亿美元，剔除不可比因素，增长15%；实际利用外商直接投资10.3亿美元，增长11.5%；城镇、农村居民人均可支配收入分别达到24190元和10130元，同口径增长9.5%、10.5%。动态指标中，地区生产总值、规上工业增加值、固定资产投资、财政收入、社会消费品零售总额、实际利用外商直接投资、城镇和农村居民人均可支配收入8项指标增速均居全省前5位。总量指标中，地区生产总值、规上工业增加值、财政收入和实际利用外商直接投资4项指标位居全省第一方阵。

  （二）抓项目增后劲，发展基础进一步夯实。

  争取国家专项建设基金项目64个，基金额度32亿元，居全省前列。新引进亿元以上项目超400个，到位资金800亿元，其中10亿元以上项目23个。全市亿元以上项目新开工340个、新竣工310个，亿元以上工业项目达产100个。以十大工程为重点的一批重大项目有新突破，汽车城建设扎实推进；健康食品城开工建设亿元以上项目23个、竣工投产17个；华塑二期、台玻电子玻璃等项目正在积极推进，博西家电产业园实施二期工程、洗碗机项目开工建设；滁马高速、宁洛高速凤阳支线建成通车，宿扬高速、滁淮高速加快施工；汊河新闸汛前竣工，滁河防洪治理近期工程正在扫尾，滁州港、沙河水库除险加固、池河防洪、淮干蚌浮段行蓄洪区调整等工程扎实推进；华汇热电、50万伏釜山变建成投运；江巷水库可研报告通过审查，合宁高铁规划线经过滁州，合连高铁首选经定远、明光线位，滁宁轻轨列入皖江城际铁路网规划近期建设项目。

  （三）抓转型强支撑，发展质量进一步提升。

  工业振兴取得新成效。净增规上企业超百户，总数达1391户。新认定国家高新技术企业49家，总数达175家，居全省第五；高新技术产业实现增加值285亿元，增长15.5%；战略性新兴产业产值507亿元，增长25.7%。市开发区跻身首批省级战略性新兴产业智能家电集聚发展基地。新增私营企业6700多户、个体工商户1.8万户，分别增长24%和45%。民企入围全省百强数连续两年居全省第三，蓝德、天康、天大进入全国民营制造业500强。

  现代农业又有新突破。粮食生产实现“十二连丰”，总产89亿斤，收购量稳居全省第一。定远获批国家农业科技园区，新增明光潘村洼和琅琊西涧省级现代农业示范区。新增家庭农场900家、农民专业合作社600家；农机化作业水平达80%，超全省9个百分点。来安成为全省首个国家级出口禽类产品示范区。

  现代服务业呈现新气象。净增限上企业31户，累计达861户。新建电商园区6个，引进企业50多家；农村淘宝多点开花，定远、明光、天长服务中心正式运营；明光成为国家电子商务进农村综合示范县，瑞飞科技荣获中国电子商务百强，淘滁网、云伙计等一批“互联网＋”企业初显身手。明光广大物流城、市开发区现代物流园等加快建设，完成投资25亿元。滁州1912休闲游、滁城西部生态游等渐成气候。全年社会融资总量超500亿元，新增贷款超160亿元，增长15.6%；新型政银担“4321”模式业务额达24亿元，居全省第四。

  （四）抓改革促开放，发展活力进一步迸发。

  着力先行先试，完成143项改革。农村土地承包经营权、不动产登记、营业执照“三证一号”等3个全省“第一证”在滁颁发；定远获批全国农村改革试验区和国家中小城市综合改革试点；天长获批全省唯一的国家集体资产股份合作制改革试点县；滁州和定远入选国家智慧城市试点，凤阳成为国家循环经济示范县试点和全省农业三项补贴合并改革试点县；全椒、南谯分别获批全国、全省第二批水生态文明建设试点城市，全市获批水生态文明建设试点城市数居全省第一。城市公立医院改革受到中央改革办充分肯定，完成市级公车改革，全面放开户籍登记、取消落户限制。进一步扩大对外开放，加快融入长三角经济圈；主动对接南京江北新区规划建设，滁州产城融合示范区已上报国家发改委待批。外贸进入全国百强市第76位，外贸实绩企业发展到900家，居全省第三。

  （五）抓建设重管理，发展条件进一步改善。

  新一轮滁州城市总体规划获批，规划面积120平方公里。强力推进“四个一”重点工程，琅琊山东麓整治、修复工程继续推进，长城影视文创城、铜矿文化旅游乐园等重点项目展露新姿；清流河二期进入全面施工阶段；古城保护性改造有序进行，内城河综合治理及南大桥、西大街桥等快速建设；明湖项目列入省“861”计划，前期各项准备就绪，正在启动一期征迁工作。洪武路、世纪大道、苏滁大道、104国道滁汊段改造等全线贯通。中心城区共实施重点项目137个，完成投资超200亿元。同步推进93个中心村建设，完成美好乡村建设项目834个、正在实施项目726个。滁城实施了限放烟花爆竹规定，来安、全椒、定远等多个县城进行了三小车整治。

  （六）抓民生办实事，发展成果进一步共享。

  全年民生支出262.5亿元。新增保障性住房2万套，完成农村危房改造1.7万户。新增城镇就业9万人。农村、城市人均保障水平分别增长12%和10%。完成省下达渔民上岸安居工程任务。老村干、老民师等七类“老字号”群体补助及时发放。市委市政府确定的十件实事取得成效，市图书馆开工建设；市一院加快推进儿童医院综合大楼和南区新院建设；内城河改造征迁房屋近1200户，基本完成清淤、截污、驳岸、游步道等工程；新增10所幼儿园，其中9所完工并招生，城区幼儿园危房改造工程已完成8所；市福利中心改扩建完成施工配套工程；市残疾人综合服务中心挂牌；改造街头游园5个、公厕12座。同时，成功举办第五届中国农民歌会及全省老年运动会等。市博物馆布展准备就绪，建成26个省级农民文化乐园示范点。推进气象现代化，建成5个国家新型气象观测站。设立环卫工人节，开展了尊重、关爱环卫工人系列活动。突出重点行业领域安全监管，事故起数和死亡人数继续双下降。

  （七）抓作风转职能，自身建设进一步加强。

  巩固党的群众路线教育实践活动成果，认真开展“三严三实”专题教育。大力推进依法行政，建立健全政府信息公开、重要文件解读、行政决策听证和专家论证、决策责任追究等制度，出台了《滁州市法治政府指标体系》、《滁州市行政机关负责人行政诉讼出庭应诉工作规定》等，着力打造法治政府。自觉接受监督，广泛听取意见。办理人大议案3件、建议141件、政协提案408件，满意和基本满意率超过98%。强力抓好作风建设，持之以恒落实中央八项规定精神，驰而不息纠正“四风”。设立市长热线服务中心，推进网络问政和阳光政务，着力打造效能政府。严格落实“两个责任”，深入推进党风廉政建设，加强公共资源管理，强化审计监督，加大违纪违法案件查处力度，着力打造廉洁政府。

  这五年，是综合实力加速跃升的五年。生产总值一年一个新台阶，从“十一五”末的696亿元增至1313亿元，年均增长11.5%，总量前移1位；规上工业增加值连跨四个百亿台阶，从296亿元增至610亿元，年均增长13.5%，总量前移3位；固定资产投资从521亿元增至1450亿元，年均增长22.7%；财政收入从90.5亿元增至230.3亿元，年均增长20.5%，总量前移3位；外贸进出口从9.1亿美元增至20亿美元，年均增长17.1%；实际利用外资从1.2亿美元增至10.3亿美元，年均增长49%，总量前移10位。工业化率由42.9%上升到48.1%，工业对经济增长的贡献率达60.7%。高新技术企业增长3.6倍，产值突破千亿元大关，居全省第三。规上企业增长1.1倍，产值超亿元企业新增138户。14个省级以上开发区主要指标实现翻番，市开发区获批国家级开发区、省级高新区；苏滁产业园一期12平方公里实现“九通一平”，完成投资超百亿。全市经济总量如期如愿冲进全省第一方阵。

  这五年，是发展质量明显提高的五年。经济结构进一步优化，三次产业比由21.3：49.2：29.5调整为17.2：53.1：29.7。先进装备、智能家电、绿色食品产值均突破500亿元，高新技术产业产值达1150亿元。全社会研发投入占GDP比重由0.6%上升至1.8%，实现全国科技进步先进市“两连冠”。发明专利申请和授权量均居全省第五。新增国家级技术中心1家、省级43家。获批为省级创新型试点城市。企业、产品、工程、管理等各方面的质量争创活动竞相开展、成果丰硕。生态建设逐步加强，新增造林231万亩，森林覆盖率31.6%，提高11.1个百分点，被评为国家园林城市、中国生态文明价值城市。安全生产连续三年获省目标管理先进单位。

  这五年，是城乡面貌发生巨变的五年。城镇化率达49%，年均提高1.47个百分点。中心城区新增建成区面积40平方公里，滁城南扩东进，新建了30多条主干道，改造、打通了20多条道路，政务新区、高铁站区、科教园区与市开发区、苏滁产业园有机联为一体；先后实施了南湖、清流河、龙池街、内城河等综合整治，完成小街小巷和老旧小区改造150多处，五年完成投资超千亿。各县市累计实施重点城建项目1500多个，完成投资1200多亿元。12个镇入选全国重点镇，秦栏、铜城跻身省宜居小镇。实施了128个省级美好乡村中心村建设，定远、凤阳、天长被评为全省美好乡村建设先进县。农村清洁工程实现乡镇全覆盖，来安农村生活垃圾“组合收运”模式被全国推广。京沪高铁、沪汉蓉高铁投入运营，高速公路通车里程全省第二；水利投资连续七年全省第一，市政公用设施投资连续五年全省前两位。

  这五年，是群众利益获得最多的五年。民生投入占财政支出比重由76.7%提高到86.6%。城镇居民人均可支配收入从15104元提高到24190元，农村居民人均可支配收入从5915元提高到10130元，年均增长12.3%和14.1%，分别高于全省0.7个、0.6个百分点。建成保障性住房14.5万套。新增城镇就业44万人。城镇五项保险参保人数达166万人次，城乡居民基本养老保险参保率达90%，在全省率先实现新农合大病保险全覆盖。学前三年毛入园率、高中阶段毛入学率分别达89.2%和96.5%，义务教育均衡发展，职教大市建设加快推进。医疗卫生水平稳步提升，每千人拥有床位数3.3张，净增1张。社会福利、社会救助、社会慈善和残疾人事业均有新进展。五年脱贫8.3万人、帮扶困难职工5.4万人次。广泛开展党的群众路线教育实践活动、“三严三实”专题教育及“三民”工程大走访活动，帮助群众解决了大批生产生活困难。在全省率先运行“阳光信访”信息系统；5个县成为全省平安县，平安村（社区）创建率达90%以上。

**二、再展宏图，同心共绘“十三五”美好画卷**

  “十三五”时期是我市全面建成小康社会的决胜期，是深化改革、加快转型的攻坚期，也是加速发展、冲刺全省第三的关键期，机遇和挑战并存，机遇大于挑战，总体上仍处于大有可为的重要战略机遇期。

  今后五年工作的指导思想是：高举中国特色社会主义伟大旗帜，全面贯彻党的十八大和十八届三中、四中、五中全会精神，深入学习习近平总书记系列重要讲话精神，认真落实“四个全面”战略布局，牢固树立创新、协调、绿色、开放、共享的发展理念，以提高发展质量和效益为中心，以加快调转促为主抓手，以增进人民福祉、促进人的全面发展为根本，稳中奋进，好中加快，能快则快，确保如期全面建成小康社会。

  市政府编制了十三五规划《纲要》（草案）。今后五年要紧紧围绕全面建成小康社会这一目标，推动“五个发展”，实现“四个翻番”，冲刺“全省第三”，争当全省承接产业转移、加快调转促、全面建成小康社会排头兵。

  ——综合实力明显增强。主要指标增速全省领先，总量再上新台阶。地区生产总值年均增长10%左右，人均地区生产总值力争达到1万美元。财政收入年均增长12%以上。

  ——转型升级成效显著。三次产业比调整到15以下：50左右：35以上。战略性新兴产业产值占规上工业比重达25%，高新技术产业增加值占规上工业比重达55%。全社会研发经费支出占GDP比重2.5%以上，科技进步贡献率50%以上。

  ——城乡统筹步伐加快。新型城镇化水平大幅提升，“多规合一”实现全覆盖，城乡布局更加优化、功能品质显著增强。“大滁城”深度融合发展，县城、重点镇基本建成现代中小城市，90%中心村建成美丽乡村。

  ——生态环境明显改善。生态文明理念贯穿经济社会发展全过程，绿色生产生活方式初步建立，生产、生活、生态空间布局科学。大气、水、土壤等污染得到有效整治，重点流域水质优良率57%以上，森林覆盖率36%以上。

  ——生活质量稳步提升。城乡居民收入增幅高于GDP增幅，人均收入达到全省平均水平，收入分配差距逐步缩小。社会事业加快发展，公共服务水平进一步提高，人民文明素质和社会文明程度明显进步。扶贫攻坚任务完成，贫困人口全部脱贫。

  实现“十三五”发展目标，重点要把握和推动五个方面工作：

  （一）聚焦厚植优势，着力推动创新发展。

  全面落实“4105”行动计划，加快构建以战略性新兴产业为先导、先进制造业为主导、现代服务业为支撑、现代农业为基础的产业发展新体系。加快省级创新型试点城市建设，推动“双创”与“互联网＋”、“中国制造2025”相结合，促进新型工业化与信息化深度融合。以推动科技创新为核心，以破除体制机制障碍为主攻方向，统筹科技、产业、市场、管理等各方面创新，推动经济增长保持中高速、产业发展迈向中高端。

  （二）聚焦统筹平衡，着力推动协调发展。

  发挥长江经济带沪宁合主轴节点城市的地缘优势，加快形成“一核两带”区域布局，谋划推进滁州行政区划调整，促进皖北振兴区域联动发展，统筹推进城镇和美丽乡村建设，逐步实现城乡居民基本权益平等化、公共服务均等化、产业发展融合化等。通过以城带乡、产城互动、城乡一体、统筹发展，着力在空间形态、交通构架、服务网络等方面突破，构建宜居宜业城镇体系。

  （三）聚焦生态保护，着力推动绿色发展。

  牢固树立绿色发展理念，完善城市功能区划，推进产业合理布局。大力发展循环经济，淘汰落后产能，抓好节能减排。坚持“五水共治”，加强水环境综合治理。加大污染防治力度，提高秸秆等综合利用水平。推进“三线三边”整治，加强农村垃圾收集转运处理，控制和减少面源污染。争创国家级生态文明先行示范区和环保模范城市、森林城市。

  （四）聚焦合作共赢，着力推动开放发展。

  把握南京江北新区等多重国家战略机遇，推进东向发展。充分发挥我市连接南京、合肥两个长三角副中心的区位优势，深度融入南京都市圈、合肥经济圈。全面参与长三角区域分工合作，构建全方位、多层次区域合作体制机制，促进要素有序自由流动、资源优化配置、市场深度融合。统筹对内合作与对外开放，坚持“引进来”、“走出去”并举，拓展开放合作空间。

  （五）聚焦民生福祉，着力推动共享发展。

  大力实施民生工程，加快发展社会事业，让人民群众生活得更加幸福、更有尊严。建立统筹城乡的基本公共服务体系，不断完善就业、基本养老、最低生活保障等制度，实现城乡居民养老保险、新农合大病保险、最低生活保障全覆盖，形成覆盖城乡的就业、救助体系。因地制宜、分类施策，众志成城实现脱贫攻坚目标。坚持教育优先发展，全面普及15年基础教育，推进义务教育基本均衡，健全现代职业教育体系，办好特殊教育。加快法治滁州建设，创新社会治理，维护社会和谐稳定。

  关于“十三五”需要进一步说明的几个问题：

  第一，关于同步实现小康社会。目前滁州人均GDP是全国的65.7%、全省的88.8%，城乡居民人均可支配收入才到全国的76.5%、全省的91.2%。而只有总量实现了翻番、人均达到了全国全省平均水平之上，这样的全面小康才是高质量的。

  第二，关于实现四个翻番、冲刺全省第三。实现四个翻番，就是实现地区生产总值、工业总产值、财政收入、城乡居民收入在2014年基础上翻番；冲刺全省第三，就是推动GDP、财政收入等主要指标冲刺全省第三位。我市只有保持10%左右的增长速度，才可望实现四个翻番、冲刺全省第三的奋斗目标。

  第三，关于调转促。“十三五”期间，全市三次产业结构要有明显优化，一产调到15%以下，三产调到35%以上，二产保持在50%左右。工业上，围绕“中国制造2025”和“工业4.0”，新上一批数字设备、数字车间、数字工厂；农业上，以示范园区引领农业现代化，不断提高农业的规模化、产业化、科技化、设施化水平；服务业上，加快电子商务、文化创意、现代金融、综合物流、旅游休闲、教育医疗、养生养老等现代服务业发展。转方式，既要转动力方式，推动主要由投资驱动向全要素综合驱动转变；也要转发展方式，推动由规模速度型增长向质量效益型增长转变。促升级，既包括产品、产业的升级，也包括总量、人均的升级，还包括企业的升级、区域的升级等。

  第四，关于扶贫攻坚。“十三五”时期全市还有13.5万农村贫困人口。通过实施脱贫攻坚十大工程，精准扶贫、精准脱贫，全市确保实现“三年脱贫、两年巩固”目标，即：到2018年底，实现定远贫困县摘帽、123个贫困村全部出列、13.5万农村贫困人口全部脱贫，再利用两年时间对少数返贫人口进行脱贫巩固。

  第五，关于重大平台。主要有：一是滁州产城融合示范区。包括苏滁现代产业园、滁宁高教科创城、汊河江北水岸科技新城三个组成部分。示范区规划已上报国家发改委待批，旨在发挥自身优势，积极呼应江北新区建设，实现无缝对接、等高对接，打造区域经济新的增长极。二是滁州西部大工业基地。涵盖定远、凤阳、明光三县市，主要立足于丰富的非金属矿产资源，以盐、石英砂、凹凸棒土三条主线延展，打造完整产业链条，推动盐化、硅基和稀土产业向千亿发展。三是滁城城南新区。包括高铁站区、科教园区、政务新区、明湖项目区四大板块，要强化领导力量、理顺管理体制、深化规划设计、完善配套功能，加速项目招引、入驻、开发。

  第六，关于重大基础设施。交通方面，主要是滁宁轻轨、合滁宁高铁、合连高铁、北沿江铁路、宁天城际二期、蚌滁宁城际、滁州支线机场、窑河复航、白塔河航道升级、滁河航运综合提升以及“四纵四横四条连接线”的滁州高速公路网等；水利方面，抓好江巷水库、山许水库、黄栗树水库改造、引江补淮等重大工程；能源方面，推进大唐电厂、泉盛化工热电联产、500千伏同乐变、1000千伏特高压输变线以及风能发电、光伏发电、生物质发电等；海关特殊监管区方面，通过招引大项目，加快建设保税物流中心、保税仓库等，全力申建海关特殊监管区。

  三、只争朝夕，全力做好今年各项工作

  2016年是“十三五”开局之年，全市经济社会发展主要预期目标是：地区生产总值增长10%左右，财政收入增长10%以上，固定资产投资增长16%，社会消费品零售总额增长12%以上，外贸进出口增长10%以上，城镇和农村居民人均可支配收入分别增长10%、11%，城镇登记失业率控制在4.4%以内，节能减排完成年度目标任务。

  重点抓好八个方面工作：

  （一）以稳定增长为首要任务，着力加快转型升级。

  推动工业扩量提质。全力抓好现有30个产业集群，重点建好市开发区智能家电基地，优先支持市开发区绿色食品、苏滁园新能源汽车、定远新型化工、凤阳硅基材料、来安轨道交通装备、天长智能仪器仪表等6个战略性新兴产业集聚发展基地。滚动实施300项重点技改项目，培育百亿元企业1家，50亿元企业4家，10亿元企业40家，亿元企业数达540家，全年净增规上企业130户以上。争取认定省级示范企业4家以上，亿元以上企业数字化装备率达55%。争取新增省级技术中心5家，省级以上创新型企业2家，高新技术产业产值占工业产值比重超过45%。

  推动服务业提档升级。突出发展电子商务。以创建国家电子商务示范市为目标，加快推进6个在建电商产业园和康佳电商、苏宁云商等项目建设，推进与阿里巴巴村淘项目合作，继续争创电子商务进农村综合示范县。大力发展现代物流。加大现代物流龙头项目招引力度，加快南京湾商贸物流园等集聚区以及鸿耀物流、广大物流等项目建设，推动滁总物流、益民物流争创5A。落实责任，推进滁城二手车、五金机电、农产品批发等几个专业市场的归行入市经营。加快发展文化旅游业。打造一批精品景点、名牌景区，重点推进琅琊山、小岗村争创5A级景区，加快长城文创、菊花博览园、农中花源、龙池街太仆寺等项目建设；充分挖掘历史文化、生态风光等特色资源，推进乡村旅游开发，打造具有地域特色的旅游品牌。着力发展现代金融。广引各类金融机构，密切政银企合作，全年社会融资总量超500亿元，新增贷款超150亿元，新增融资担保额超55亿元。加快企业上市，提高上市公司再融资能力，力争新增首发上市企业1家，新增“新三板”挂牌企业10家以上。注重防范金融风险。稳定发展房地产业。积极化解房地产库存，扩大货币化安置比例，建立购租并举的住房制度，把公租房范围扩大到进城落户、务工人员，扩大住房公积金制度覆盖面。

  推动县域经济多极并进。完善县域城镇布局，引导人口加快集聚和资源要素合理配置，支持有条件的县城、中心镇发展为中小城市。把园区作为县域工业发展的主要载体，提高投资强度，推进产业集中集群集约发展。大力发展特色产业，定远、凤阳、明光要重点围绕资源深加工，提高资源综合利用效率，用循环经济理念建设西部大工业基地；天长、来安、南谯、全椒等沿苏经济带要抓住江北新区建设机遇，重点围绕汽车装备、智能家电、健康食品、新型材料产业实施招大引强。加快发展镇域经济，镇当城建，以炉桥、大墅等为战略支点，打造产业园区，承接省会辐射。

  （二）以扶贫攻坚为当务之急，加速改变农村面貌。

  全力推进精准扶贫。全面开展建档立卡回头看活动，摸清摸实底数；在此基础上，逐村逐户找出致贫原因，对症施策。健全落实“领导包县、单位包村、干部包户”等帮扶机制。实施脱贫攻坚十大工程，综合利用产业扶持、转移就业、易地搬迁、教育支持、医疗救助、社保兜底等途径。市财政从今年起每年切块安排5000万元扶贫资金，同时整合涉农等相关资金5000万元以上用于扶贫。首战必胜，确保2016年47个贫困村脱壳、4.48万贫困人口脱贫任务胜利完成。

  大力发展现代农业。加快发展适度规模经营，全年新增土地流转面积20万亩以上，新增家庭农场900家、农民专业合作社400家。推进农业产业化经营，新增销售收入超亿元龙头企业15家、超20亿元1家，完成农产品加工产值700亿元。加快农业物联网建设，新增示范点10个。推进农机农技农艺深度融合，开展粮食生产全程机械化示范县创建，建立示范点8个。鼓励社会资本、工商资本投入现代农业，整合涉农项目资金，引导资本、技术等要素向园区集聚，全年新创建市级以上现代农业示范园区10家以上，力争凤阳获批国家现代农业示范区。大力推动农业与工业、农业与旅游、农业与商贸、农业与互联网、农业与新型城镇化等融合发展、跨界发展。

  加快建设美丽乡村。天长整县域开展美丽乡村建设试点。全面推进中心村建设、自然村环境整治，优先抓好50个乡镇政府驻地的环境整治和中心村建设。切实保护好农村的生态环境、自然水系、村庄肌理、田园特色、传统风貌，继续打造精品示范线和精品区块。

  （三）以改革开放为强大动力，奋力抢占发展先机。

  进一步抓好重点领域改革。农村综合改革上，全面完成农村土地承包经营权确权登记颁证，做好农村集体土地确权登记发证工作，深化农村集体产权制度改革、小型水利工程管理体制改革、供销社综合改革，实施国有林场改革。经济体制改革上，推进国有集体企业改制扫尾，发展混合所有制经济。加快财税体制改革，创新财政支持方式，完善事权和支出责任匹配的财政体制。深化投融资体制改革，积极推行与社会资本合作（PPP）。加速金融改革创新，推进市县政策性融资担保体系建设。行政体制改革上，简政放权，深化商事制度改革，实施“先照后证”，完善“一照一码”；推进行政审批相对集中改革，建立一门受理、并联审批和多证联办的服务模式；整合建立统一的公共资源交易平台，推进市县一体化试点。社会事业体制改革上，实质性开展县域中小学教师“无校籍管理”改革试点，推进学校用人机制和收入分配制度改革。推进城乡均等化的公共就业创业服务体系建设。加快公立医院改革。新型城镇化改革上，落实户籍制度改革各项任务，实行居住证制度，推进城镇基本公共服务向常住人口全覆盖。在苏滁园、汊河、乌衣、炉桥等地探索新型城镇化新路径。推进定远国家中小城市综合改革试点。

  进一步强化区域合作。深度融入长三角，加快产业、交通、市场、通关及公共服务等一体化进程。强化“大江北”观念，对毗邻江北新区区域进行系统规划，推动整体融合、功能互补、错位发展，支持金牛湖、汊河、乌衣等节点地区率先融入、率先突破，打造协同发展的战略平台；推动滁宁合作项目化，促进城市规划、基础设施、产业转移、公共服务等方面共建共享，构建滁宁半小时生活圈。推进与合肥一体化，深化两地在基础设施、产业布局、要素市场等方面合作。争取省政府延续皖江示范区相关政策，落实省促进皖北发展政策，积极参与淮河流域治理和淮河生态经济带创建。提升外贸发展水平，天长海关监管中心正式运行，提升保税仓库运营水平，申建滁州无水口岸、保税物流中心。

  进一步激活民营经济。降低准入门槛，减轻企业负担，加强新型政银担合作，发挥续贷过桥资金作用，推进“税融通”。加快创客空间等新型科技孵化器及小微企业创业基地等载体建设。全年新增私营企业5000户、个体工商户1.7万户。深化与知名民企合作，新增5亿元以上民企合作项目30个以上。

  （四）以招商引资为第一抓手，狠抓项目落地生根。

  突出抓好招大引强。突出引资引技引智一体，高位推进，进一步发挥县干招商小组作用，大力推进精准招商、以商招商、产业链招商、驻点招商、代理招商、网上招商，主攻重点区域，力争招引一批行业龙头企业和关键配套企业，做大产业规模，加粗产业链条，形成集群效应。务求在新能源汽车、智能家电、大健康产业的大项目招引上取得突破。全年新引进亿元以上项目400个，其中10亿元以上项目超过30个，年到位资金880亿元；力争400个项目列入省重点项目计划。

  加大项目推进力度。每个项目都要落实责任单位、责任人，重大项目必须由市县领导牵头抓；项目推进过程中的协调、调度要经常化、规范化，协调、调度要解决问题、记录在案；盯紧具体项目、盯紧重点目标人、盯紧关键环节、盯紧竞争对手，力求抓一件成一件；严肃认真对待项目考核，注重考核结果的运用，兑现奖惩。

  完善园区配套设施。优化园区发展规划、产业定位，培育主导产业、特色产业，促进产业集群、企业集聚、资源集约。优先解决园区水、电、路、气、通信、排污等必要的生产设施配套问题。着力提升公共服务功能，加强公交、学校、医院、银行、邻里中心等配套设施建设。市开发区要加快推进“三次创业”。苏滁产业园要在20亿元以上大项目和3亿美元以上外资项目上实现突破，全年新签约、新开工、新投产亿元以上工业项目分别达20个、15个、10个以上。天长开发区争取成为国家级开发区和转型升级示范区，全椒开发区扩区争取获批，汊河开发区争创省级战略性新兴产业集聚发展基地，推进凤阳·宁国、定远·肥东、明光·肥西合作共建园区。

  （五）以城乡统筹为主要路径，提升建设管理水平。

  中心城区要提升提神。推动滁城由外延扩张式向内涵提升式转变。一是提升规划水平。实施新一轮城市总体规划，强化滁来全规划衔接。严格规划实施，切实发挥好规划的引领和调控作用。尽快编制实施海绵城市规划、管廊规划和水生态体系规划。加强城市设计，强化城市轮廓线、标志性建筑等建设管理，注重特色塑造，挖掘城市历史文化内涵。二是推进重点工程。以“四个一”工程为重点，实施好总投资超50亿元的44个重点建设项目。琅琊山以创建5A级景区为统领，完成核心景区整治；规划建设景区路网，推进重大文、旅、休、养类项目落地、建设。清流河基本完成二期工程建设，并做好一、二期工程的衔接调整，着手谋划三期工程。明湖工程全面启动，加速大坝建设和湖区整理、路网建设、湿地景观营造等，2017年汛前下闸蓄水。古城继续稳准推进保护改造提升，注重文脉延续，彰显“滁”字山、水、城的特色。三是补齐城市短板。推进城南新区学校、医院、菜场、超市等建设。加强住宅小区综合治理，健全物业服务、监管机制，着力解决小区顽症，处置问题楼盘。四是加强城市管理。健全管理重心下移、执法集中统一、建管养分离、网格责任到人的长效机制，深入开展市容环境综合整治行动。推进网络共建共享，加快智慧城市建设。整治占道经营、乱停乱放，改造瓶颈路段和节点，扎实开展好违建整治专项行动。推进文明城市、卫生城市、森林城市、环保模范城市、历史文化名城等五城联创。

  美好城镇要做精做强。遵循产城融合、新老并重、建管一体的基本原则，继续把县城建设好。进一步提升规划设计水平，完善配套服务功能，把握好房地产开发节奏；进一步加强城市管理，使“整洁”全覆盖、常态化。推进小城镇建设，着力打造乌衣、汊河、炉桥、临淮、铜城、大墅等产业新城，藕塘、半塔、女山湖等旅游小镇；选择汊河、炉桥、秦栏等地开展撤镇设市试点。

  基础设施要抓主抓重。推进滁宁轻轨、宁天城际二期、合连高铁、合滁宁高铁等重大项目前期工作，务求合宁高速扩建、滁天高速、来六高速连接线等开工。加速滁淮高速、滁州港、农村道路畅通工程等项目建设。深入比选场址，推进大唐电厂建设。加快农村电网改造，完成220千伏团山变、贺庄变建设，推进中海油等天然气管道项目。江巷水库启动征迁并开工建设，完成沙河水库除险和滁河近期治理等工程，除险加固中小型水库102座。

  （六）以蓝天碧水为最大心愿，全力保护生态环境。

  加强生态建设。完成森林增长工程任务，深入开展绿色滁州行动，重点实施城市景观、交通干道、工业园区、农田林网等绿化工程，新增造林16.8万亩。加大生态市、生态县、生态镇村创建力度。开展生态修复，完成50处关闭矿山环境治理任务。创建西涧湖、沙河集、黄栗树三大水库生态保护区，推行以河长制为核心的水质保护责任制。推进节能减排。严把新上项目能评关、环评关，淘汰落后产能，完成省下达的节能减排目标任务。划定滁城高污染燃料禁燃区，加大黄标车淘汰力度，继续开展秸秆禁烧行动。加快建设污水处理厂及污水管网，推广农村生活垃圾“组合收运”模式。高效利用资源。加快划定永久性基本农田，加速高标准基本农田建设。积极有序处置“僵尸企业”，腾笼换鸟，继续开展矿山整治，提高土地矿产利用效率。坚守水资源开发利用控制、用水效率控制、水功能区限制纳污“三条红线”。抓好城市矿产示范基地、循环经济示范园区、清洁化园区建设。扶持发展新能源、节能环保产业，大力推广资源节约、替代和循环利用技术。

  （七）以改善民生为根本目的，切实推进普惠共享。

  强化社会保障。做好大学毕业生、复退军人、城镇就业困难人员等重点人群就业工作，新增城镇就业4.2万人。适时实施机关事业单位养老保险，推进全民参保登记，推广应用社会保障卡，扩大社会保险参保覆盖面。推进城镇居民医保和新农合两项制度“二合一”。进一步提高城乡低保标准，为五保对象购买重大疾病商业保险。加快住房保障体系建设，改造棚户区8600户。积极引导社会资本兴办养老机构，在引进中高端养老养生项目上有新突破。

  繁荣社会事业。办好学前教育，全面完成学前教育三年行动计划；均衡发展义务教育，力争全部通过省级复评和国家评估验收；加快发展现代职业教育，推进职业技术学院创建地方技能型高水平大学，启用城市职业学院新校区，健全中职、高职、本科有机衔接的培养机制；启动特教学校建设。实施社区卫生服务机构提档升级工程，市一院南区投入使用，完善市二院功能，加快市中西医结合医院扩建；积极稳妥有序落实“全面二孩”政策。完善公共文化服务体系，市博物馆年内开馆，推进市图书馆及文化艺术中心建设；新建10个省级农民文化乐园、10个乡镇综合文化服务中心。加强气象防灾减灾能力建设，争取新一代天气雷达建设项目落户滁州。加强国防动员，推动军民融合深度发展。加快发展体育事业、体育产业。支持工会、共青团、妇联等群团组织开展好活动，统筹抓好外事、侨务、对台、民族宗教、统计、物价、编制、档案、保密、人防、地震、科协、老干部、地方志、红十字会等工作。

  创新社会治理。加强基层基础工作，推进社区服务管理信息化，深入开展和谐社区创建活动。完善多元化矛盾纠纷调处机制，推动矛盾及时就地化解。抓好立体化社会治安防控体系建设，依法打击各类违法犯罪活动。健全舆情管控机制，维护网络秩序和安全。完善食品药品生产、流通、消费全环节监管体系，保障群众饮食用药安全，争创省食品药品安全市。深刻汲取各类事故教训，强化红线意识和底线思维，严格落实安全生产责任制，坚决遏制重特大事故发生。强化应急管理，做好公共安全风险评估和防控。

  办好为民实事。今年市政府承诺的十件实事为：滁州中合民生农产品批发市场投入运营、迁建改造4个农贸市场；改造滁城棚户区2000户和扬天小区、润芳园、清风园、建工大院4个老旧小区；建设市植物园；实施南谯路（明光路至花园路段）畅通工程；建设城北客运站；加强滁城养犬管理；加强滁城老旧电梯维护和应急救援平台建设；开工建设市第三污水处理厂及配套管网项目；建成滁宁公交换乘中心；增加滁城公交、出租运力和公共自行车布点。

  （八）以作风建设为永恒主题，打造人民满意政府。

  增强看齐意识。认真学习《党章》、《条例》和习近平总书记系列重要讲话精神，不断提高政治觉悟和思想定力。严守政治纪律和政治规矩，更加坚定地把“四个自觉”贯彻到修身做人、用权律己、干事创业的各个方面。以党中央为最高标杆，以中央要求为行动指南，自觉主动向中央看齐，做政治上的明白人、落实上的带头人，打造政治过硬的政府班子。

  严格依法行政。依法履行政府职能，切实做到“法定职责必须为、法无授权不可为”。严格执行重大行政决策程序规定，不断提高依法、科学、民主决策水平，确保行政决策制度科学、程序正当、过程公开、责任明确。自觉接受人大、政协和社会各界监督，主动听取民主党派、工商联、无党派人士和人民团体意见建议。认真办理人大议案、建议和政协提案。

  强化责任担当。牢记“四有”要求，进一步强化担当意识，自觉扛起责任，推动重大决策、重点工作、重要项目落实。简化办事流程、提高服务效能，推进四级政务服务信息系统一体化，开展国家级政务服务综合标准化试点。加强行政监察和审计监督，坚决纠正不作为、乱作为，坚决克服懒政、怠政，坚决惩处失职、渎职。

  坚持从严治政。严格遵守廉洁从政各项规定，深入落实党风廉政建设责任制和“一岗双责”，完善政府系统惩防体系，强化权力运行监督，把权力关进制度的笼子。坚持无禁区、全覆盖、零容忍惩治腐败，严肃查处发生在重点领域、关键环节和群众身边的腐败案件。持之以恒反“四风”、改作风，厉行勤俭节约，切实做到为民、务实、清廉。
